# High yield and ultrafast sources of electrically triggered entangled-photon pairs based on strain-tunable quantum dots

**DOI:** 10.1038/ncomms10067

**Published:** 2015-12-01

**Authors:** Jiaxiang Zhang, Johannes S. Wildmann, Fei Ding, Rinaldo Trotta, Yongheng Huo, Eugenio Zallo, Daniel Huber, Armando Rastelli, Oliver G. Schmidt

**Affiliations:** 1 Institute for Integrative Nanosciences, IFW Dresden, Helmholtzstrasse 20, 01069 Dresden, Germany; 2 Institute of Semiconductor and Solid State Physics, Johannes Kepler University Linz, Altenbergerstrasse 69, 4040 Linz, Austria; 3 Material Systems for Nanoelectronics, TU Chemnitz, 09107 Chemnitz, Germany

## Abstract

Triggered sources of entangled photon pairs are key components in most quantum communication protocols. For practical quantum applications, electrical triggering would allow the realization of compact and deterministic sources of entangled photons. Entangled-light-emitting-diodes based on semiconductor quantum dots are among the most promising sources that can potentially address this task. However, entangled-light-emitting-diodes are plagued by a source of randomness, which results in a very low probability of finding quantum dots with sufficiently small fine structure splitting for entangled-photon generation (∼10^−2^). Here we introduce strain-tunable entangled-light-emitting-diodes that exploit piezoelectric-induced strains to tune quantum dots for entangled-photon generation. We demonstrate that up to 30% of the quantum dots in strain-tunable entangled-light-emitting-diodes emit polarization-entangled photons. An entanglement fidelity as high as 0.83 is achieved with fast temporal post selection. Driven at high speed, that is 400 MHz, strain-tunable entangled-light-emitting-diodes emerge as promising devices for high data-rate quantum applications.

## Abstract (web summary)

Quantum communications require sources of entangled photons. Electrically triggered sources usually suffer from low entangled-emission efficiency. Here, the authors use piezoelectric strains to tune the fine structure of quantum dot emitters, and increase the entanglement probability and fidelity.

## Graphical abstract

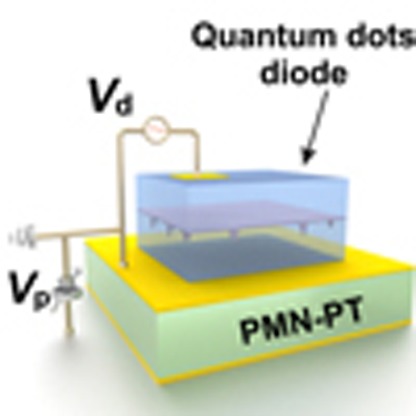

A source of triggered entangled photon pairs is a fundamental element in quantum information science and plays a key role in a number of photonic quantum technologies such as linear quantum computation^1^, quantum teleportation^2^ and quantum relays^3^. So far, generation of polarization-entangled photon pairs is mostly obtained through spontaneous parametric downconversion^4^ and four-wave mixing^5^ in nonlinear optical media. At present, these sources are optically driven with lasers, which increases the complexity of the systems. In addition, the nonlinear optical process occurs randomly so that the emission of entangled photon pairs is probabilistic. This results in the generation of zero or multiple entangled-photon pairs in most excitation cycles and unavoidably limits the success of realizing deterministic photonic quantum technologies.

These complications could be alleviated by employing solid-state quantum systems such as colour centres in diamond^6^, intrinsic defects in silicon carbide^7^ and semiconductor quantum dots (QDs)^8^. They exhibit atomic-like optical transitions and therefore allow the generation of deterministic single photons. Most importantly, they can be easily embedded in a light-emitting diode so that electrically driven single-photon emission from these systems can be realized^9^,^10^,^11^. However, to date, colour centres in diamond and intrinsic defects in silicon carbide are limited to emit single photons and the generation of entangled-photon pairs from these two systems has not been demonstrated yet. In contrast, semiconductor QDs are proven candidates for generation of deterministic entangled-photon pairs. In the last decade we have witnessed tremendous advancements in the field, for instance, ultrabright^12^,^13^ and highly indistinguishable polarization-entangled photons^14^ and time-bin entangled photons^15^ have been demonstrated successfully with semiconductor QDs. Remarkably, a robust and compact entangled-light-emitting-diode (ELED)^16^ based on semiconductor QDs has been realized, which represents a significant progress in the field of entangled-photon sources. Furthermore, the recent seminal realization of quantum teleportation with ELEDs^17^,^18^ has indicated the desirability and the ultimate feasibility of using such ELEDs for various future quantum applications.

In analogy to the cascade emission in atomic systems^19^, electrical injection of electrons and holes into an ELED containing QDs switches on the radiative decay of biexciton (*XX*) to the exciton (*X*) and finally to the ground state (*0*)^20^. The two ideally degenerate intermediate *X* states (spin±1) ensure emission of polarization-entangled photon pairs and the two-photon quantum-mechanical state can be expressed with the Bell state 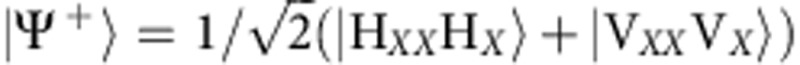 (H and V denote the orthogonally horizontal and vertical polarizations). In real QDs, however, a reduced structural symmetry due to the anisotropy in strain, composition and shape results in the appearance of an energetic splitting between the two bright *X* states, the so-called fine structure splitting (FSS)^21^. In the presence of a FSS, the entangled state evolves over the *X* lifetime and the time-averaged fidelity to the Bell state reveals classical correlations among the emitted photons. High fidelity to the state |Ψ^+^〉 can be only observed by temporal post selection of the emitted photons that, however, results in a strong reduction of the brightness of the quantum source. Therefore, the FSS in an ELED containing QDs is the key parameter determining the quality of the entangled-photon pairs. Recent work has shown that in standard self-assembled QDs^22^, the probability of finding QDs with a FSS smaller than the radiative linewidth of the *X* emission (1 μeV) is lower than 10^−2^. This finding implies that as-grown QDs are still impractical for scalable quantum networks. For example, it was reported that only one QD per ELED shows an FSS small enough for entangled-photon generation^16^. In this context, the real potential of ELEDs for entangled-photon generation can be harnessed only when a tight control over the FSS is achieved.

The FSS of semiconductor QDs can be suppressed or tuned to zero via the application of either a vertical electric field^23^,^24^ or a combination of strain and electric field^25^. The main drawback of these approaches is the difficulty of using the electric field to control the FSS and to inject carriers simultaneously, as in ELEDs. Other techniques such as thermal annealing^26^ and in-plane magnetic field^27^ would be potentially compatible with the ELEDs, but the former requires a lengthy procedure and the latter bulky setups, thus rendering a practical implementation inconvenient. Recent theoretical works (although not experimentally realized so far) suggest that the FSS can be eliminated using solely a well-controlled strain field^28^,^29^. Using piezoelectric-induced strains to engineer the properties of ELEDs would be highly desirable, because this fully electro-mechanically controlled tuning knob would allow the problems related to the FSS in ELEDs to be overcome.

Here we experimentally demonstrate such a quantum device by integrating ELEDs onto a piezoelectric actuator featuring giant piezo-electric response and capable of delivering well-defined anisotropic strain fields. With this device—which we call strain-tunable ELED (ST-ELED)—we show that the FSS of QDs can be tuned effectively with the elastic strain fields without affecting the electrical injection of the operation of the ELEDs. Up to 30% of the QDs are tuned to be suitable for the generation of entangled-photon pairs (more than an order of magnitude more than in previous devices^16^) and a high operation speed for an entangled-photon source is achieved, that is 400 MHz. This set of properties paves the way towards the exploitation of ELEDs in high data-rate entangled-photon applications involving a large number of quantum emitters.

## Results

### Self-assembled QDs in strain-tunable diode devices

**Figure 1:**
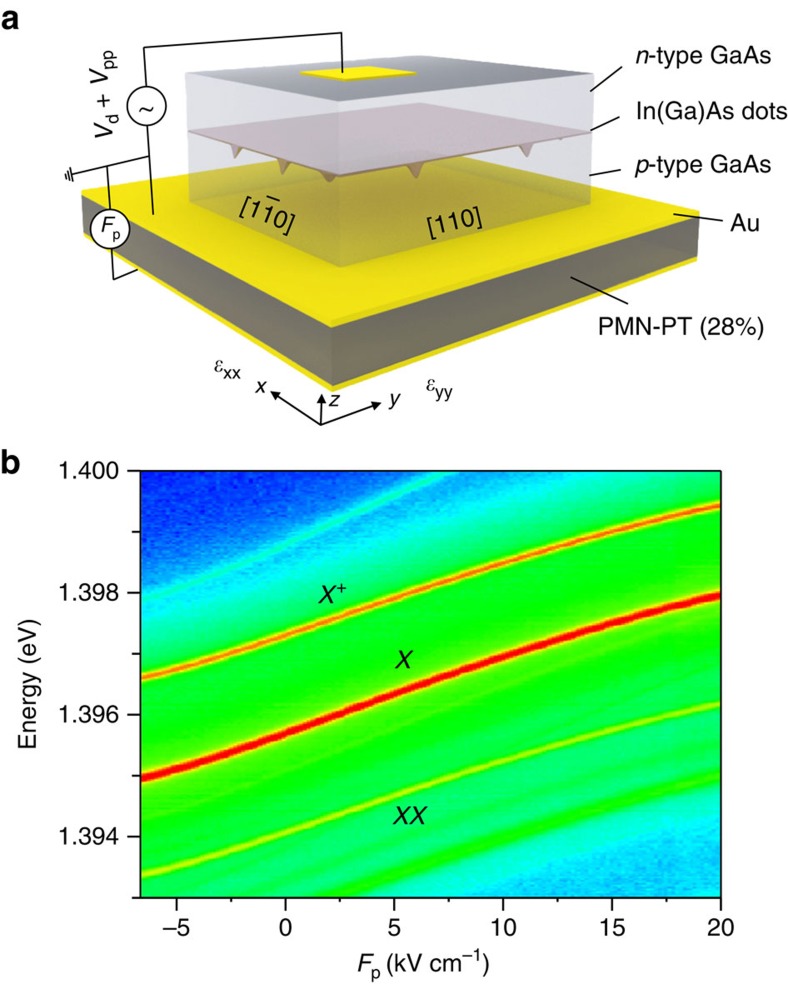
Strain-tunable entangled-light-emitting diode. (**a**) Sketch of the diode structure. Different from previous works, the PMN-PT top surface has (011) orientation, which imposes large anisotropic strain fields with well-defined orientation onto the overlying ELED. (**b**) EL from a single QD in an ST-ELED versus electric field *F*_p_ applied to the PMN-PT actuator.

The ST-ELED studied in this work is schematically shown in Fig. 1a. A 440-nm-thick *n-i-p* nanomembrane containing InGaAs QDs is integrated onto a 0.3-mm-thick [Pb(Mg_1/3_Nb_2/3_)O_3_]_0.72_[PbTiO_3_]_0.28_ (PMN-PT) single piezoelectric crystal. The detailed fabrication process is described in Methods. Different from previous works dealing with strain tuning of QDs via PMN-PTs^30^,^31^, the actuator used here has pseudo-cubic cut directions [100], [0-11] and [011], denoted by *x*, *y* and *z* axis, respectively. When the PMN-PT is poled along the *z* axis, in-plane strain fields with normal components *ε*_xx_ along the *x* axis and *ε*_yy_ along the *y* axis with opposite sign can be transferred to the nanomembrane. Accounting for its relevant piezoelectric coefficients *d*_31_∼+420 pC N^−1^ along the *x* axis and *d*_32_∼−1,140 pC N^−1^ along the *y* axis^32^, the in-plane anisotropy is estimated to be *ε*_xx_≈−0.37*ε*_yy_. The large and well-controlled strain anisotropy and the broad range of attainable strain magnitudes are unique and turned out to be vital in our work.

### Tunable electroluminescence under applied strain fields

Apart from the strain fields, electrical contacts are arranged in such a way that electrical fields can be independently applied across the diode and the PMN-PT actuator. By biasing the diode and applying a variable electric field (*F*_p_) to the PMN-PT simultaneously, energy-tunable electroluminescence (EL) from a single QD is produced, as shown in Fig. 1b. According to the power and polarization-resolved measurements the observed EL lines are ascribed to exciton (*X*), biexciton (*XX*) and charged exciton emission (*X*^+^), respectively. As the magnitude of *F*_p_ is varied, all the emission lines shift in energy. In a first approximation, this shift is due to the strain-induced change of the energy bandgap of the material, which in turn is proportional to the volumetric strain *ε*_tot_=*ε*_*xx*_+*ε*_*yy*_+*ε*_*zz*_ at the QD position. For in-plane stress and cubic materials, *ε*_tot_ is given by 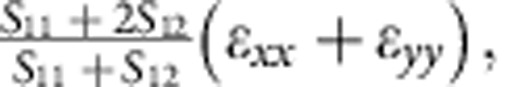
which, in the present case, is 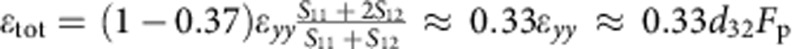 (with *S*_*ij*_ the compliance coefficients of the host material, see Supplementary Note 1). As *ε*_tot_ has the same sign as *d*_32_, which has a relatively large magnitude and is negative, we expect a positive *F*_p_ to induce a compressive strain, which results in a blue shift of the EL, whereas a negative *F*_p_ induces a tensile strain, which results in a red shift. We also note that a total energy shift of about 2.5 meV is achieved as *F*_p_ is varied from −6.7 to 20 kV cm^−1^.

### Changes in polarization and FSS with the strain fields

**Figure 2:**
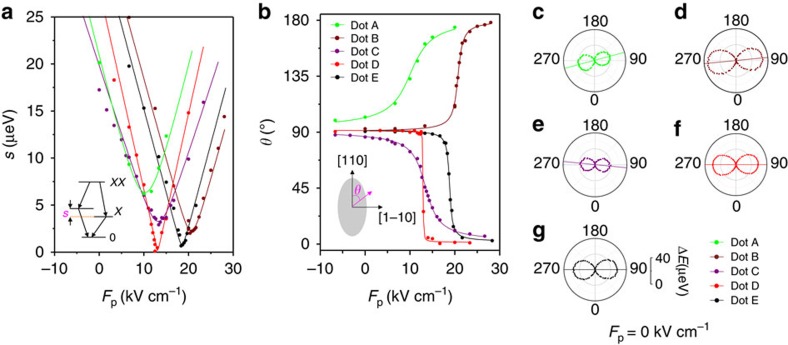
Strain-induced change of fine structure splitting and exciton polarization angle. (**a**,**b**) Representative variation of *s* and the polarization direction *θ* of the high-energy component of the exciton as a function of *F*_p_ for five QDs. The insets show sketches of biexciton cascade and the orientation of the exciton polarization. (**c**–**g**) *s*_0_ and *θ*_0_ for the five studied QDs at *F*_p_=0 kV cm^−1^. In the polar plot 0° corresponds to the [110] axis and 90° to the [1-10] crystal axis of the GaAs nanomembrane.

To control the FSS of the QDs embedded in the diode, the crystal axes [1-10] and [110] of the GaAs nanomembrane were carefully aligned along the *x* and *y* axes of the PMN-PT actuator, respectively (see Fig. 1a). Representative plots of the FSS (*s*) of different QDs as a function of *F*_p_ are shown in Fig. 2a. Although the emission energy shift is only about 2.5 meV as *F*_p_ is varied from −6.7 to 28 kV cm^−1^ as a consequence of the strong strain anisotropy (and thus relatively small *ε*_tot_), the FSS is tuned over a broad range from 30 to 0 μeV. Away from the minimum FSS, all studied QDs exhibit an approximately linear change in the FSS with *F*_p_ at a rate of about 2.0 μeV kV^−1^ cm^−1^, which is about nine times larger than what has been reported for vertical electric fields^23^. In addition to this drastic change in FSS, we also observe rotations of the exciton polarization angle *θ*, that is, the polarization direction of the high-energy line of the exciton with respect to the [110] direction of the GaAs nanomembrane (see Fig. 2b). At the largest available tensile (compressive) strain, *θ* tends to be directed along the [1-10] ([110]) direction for all QDs. Furthermore, we note that the above tuning behaviour is mainly determined by the exciton polarization angle at zero strain fields (*θ*_0_) with respect to the predefined direction of the strain^29^,^33^. Experimentally, this polarization angle can be extracted from Δ*E*=|*E*(*φ*, *F*_p_=0)−*E*_min_|, where *φ* is the polarization direction selected by our polarization analyser (which varies from 0° to 360°), *E*(*φ*, *F*_p_=0) is the relative position between *X* and *XX* as a function of *φ* and it can be directly extracted from a polarization-resolved EL measurement, and *E*_min_ is the minimum energy of *E*(*φ*, *F*_p_=0; see Supplementary Fig. 1). Figure 2c–g show Δ*E*(*φ*, *F*_p_=0) for the five studied QDs. The initial FSS *s*_0_ and *θ*_0_ are represented by the magnitude and orientation of the lobes in each polar plot. For the dot A and B, *θ*_0_ are 102.0°±0.4° and 92.7°±0.2° (the numbers quoted are the mean±s.d. and the same definition is applied below), respectively, suggesting positive deviations of *θ*_0_ from the strain *x* axis. Consequently, as *F*_p_ is increased, the polarization angle *θ* rotates counterclockwise and the FSS experiences a finite lower bound *s*_min_=7.2±0.2 and 2.2±0.1 μeV (the green and wine curves in Fig. 2a,b). For dot C, *θ*_0_ is found to be 86.1°±0.4°, which suggests a negative deviation from the strain *x* axis. Thus, a clockwise rotation of *θ* over *F*_p_ is observed, together with an *s*_min_=4.2±0.2 μeV (the purple curve). For the QDs D and E, *θ*_0_ is found to be 90.4°±0.3° and 90.6°±0.3° respectively, which indicate exact alignment of *θ*_0_ with the strain *x* axis. The polarization angles for both QDs rotate clockwise as the strain is varied and this implies that their polarization angles at zero strain fields are oriented at angles slightly <90°. It should be noted that this difference from the observed values (larger than 90°) is ascribed to the limited alignment precision of the polarizer (within a few degrees)^29^. Most importantly, owing to this exact alignment between the exciton polarization angle *θ*_0_ and the strain axes for dot D and E, their FSS can be reduced well below 1 μeV and *s*_min_ is found to be 0.30±0.25 and 0.60±0.20 μeV, respectively. By simply treating the anisotropic strain as an effective uniaxial strain^28^ (see Supplementary Note 2), the behaviour of *s* and *θ* are well fitted (solid lines in Fig. 2a,b). Noticeably, the minimum reachable FSS value, following the form *s*_min_=*s*_0_|sin(2*θ*_0_)|, is determined by *s*_0_ and *θ*_0_ (see Supplementary Table 1). Even more, the *s*_min_ predicted before varying the strain fields are 8.18±0.20, 2.34±0.21, 3.3±0.18, 0.24±0.32 and 0.76±0.15 μeV for dot A, B, C, D and E, respectively, which show excellent agreement with our experimental data. From our experimental observations and theoretical analysis, it is clear that, to cancel the FSS with our external strain fields, the strain principal axes should be as close as possible to the polarization angle of QDs at zero applied stress.

### Quantum state tomography measurments

**Figure 3:**
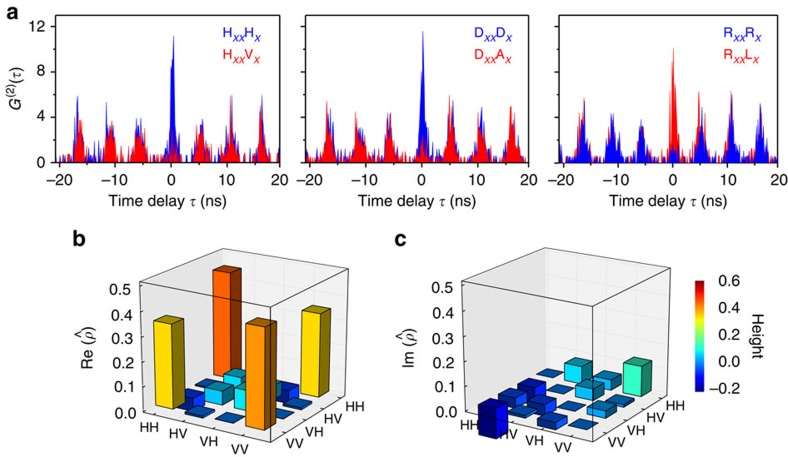
ST-ELED as source of polarization entangled photons. (**a**) Co-polarized (blue) correlation and cross-polarized (red) correlation counts (*G*^(2)^(*τ*)) for a QD in an ST-ELED excited with electrical pulses with 185.2 MHz repetition rate, measured in the rectilinear, diagonal and circular bases. Representative density matrix 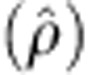: (**b**) real part and (**c**) imaginary part, which are reconstructed with 16 coincidence counts integrated in a 1.8-ns temporal window centred at 0 delay time.

The ability to tune the FSS of the QDs to zero allows us to investigate the capability of the ST-ELED to generate polarization-entangled photon pairs without the aid of post-filtering techniques^34^,^35^. Figure 3a shows the polarization resolved co- and cross-polarization correlation between the *XX* and *X* photons emitted by dot E under electrically pulsed excitation and tuned to a FSS of 0.60±0.20 μeV (at *F*_p_=18.7 kV cm^−1^). The periodic correlation peaks with well-separated temporal distance of 5.4 ns arise from the chosen repetition rate of 185.2 MHz. Importantly, for co-polarized two-photon, strong correlations are observed in linear (HV) and diagonal (DA) bases, while strong anti-correlations are observed in circular basis (RL), as expected for the photon pairs emitted in the maximally entangled Bell state |Ψ^+^〉. To quantify the degree of polarization entanglement, we have reconstructed the two-photon density matrix by performing quantum state tomography measurements, as described in ref. 36. Sixteen polarization correlation measurements were performed and the density matrix is reconstructed using a maximum likelihood estimation. The imaginary part and the real part of the density matrix are displayed in Fig. 3b,c. The outer off-diagonal elements in the real part of the density matrix reveal a high probability for a superposition of the two-photon wave function, being a clear signature of polarization entanglement^36^. Specifically, the density matrix can be used to quantify the degree of entanglement by extracting the tangle *T*, the concurrence *C*, the largest eigenvalue *λ* and the Peres criterion *P*. We find *C*=0.688±0.040 (>0), *T*=0.474±0.055 (>0), *λ*=0.795 (>0.5) and *P*=−0.30±0.02 (<0). All these tests exceed the classical limit, proving that the quantum state obtained in our experiment is highly entangled. Using the largest eigenvalue, we are able to determine the most probable state of the system: 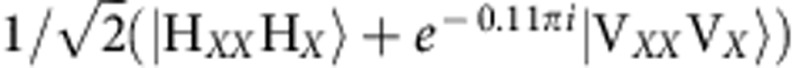, in which the presence of the phase *ϕ*_0_=−0.11*π* is likely to be due to the reflection at the beam splitter^37^ and the residual FSS 38. As a consequence, the fidelity to the maximally entangled Bell state |Ψ^+^〉 is found to be *f*^+^=0.766±0.051.

### High yield of QDs for entangled-photon generation

**Figure 4:**
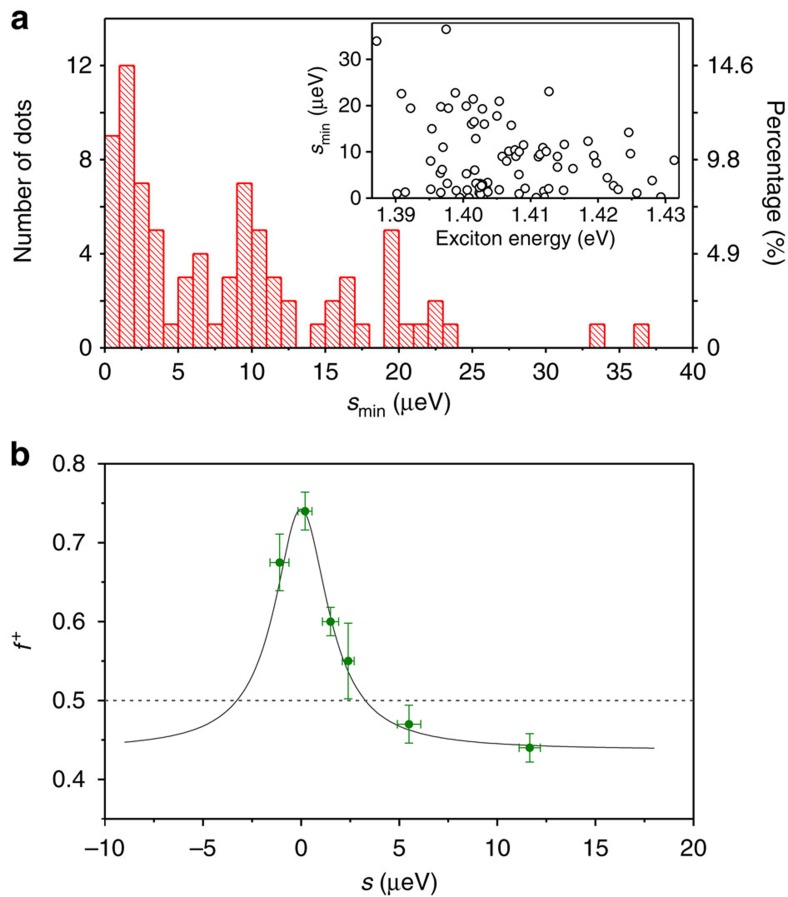
Statistical investigation of the minimum FSS and dependence of the entanglement on the value of FSS. (**a**) Histogram of the distribution of the minimum FSS (*s*_min_) tuned by the externally induced strain fields in the ST-ELED device and the right *y* axis corresponds to the histogram probability. The inset is a scatter plot of *s*_min_ as a function of the *X* emission energy and *s*_min_ shows no energy dependence. (**b**) Fidelity (*f*^+^) as a function of *s* dynamically tuned by the anisotropic strain fields and the solid line is Lorentzian fit with a full width at half maximum of 3.3±0.2 μeV. The dashed line indicates the classical value of 0.5 and the error bars are defined as the s.d.

Having demonstrated generation of entangled-photon pairs from our ST-ELED, we present one of the most important results of our work, that is, the capability of anisotropic strain fields to tune about 30% of the QDs for entangled-photon emission. A statistical study from 82 randomly selected QDs revealed that the majority of QDs in our ST-ELED device have *θ*_0_ oriented close to the [1-10] crystal axis (see Supplementary Fig. 2). Therefore, the alignment of the strain axes parallel (or perpendicular) to the [1-10] crystal axis of the GaAs ensures frequent observation of QDs with low value of *s*_min_. Figure 4a shows the statistical investigation of *s*_min_, falling into a range of 0 to 40 μeV. Remarkably, nine QDs have *s*_min_ <1 μeV, which is of the order of the homogenous linewidth of the *X* emission. Therefore, 11% of dots have sufficiently small FSS for strong entanglement in our device (see Supplementary Note 3, Supplementary Fig. 3 and Supplementary Table 2 for the entanglement results measured for other dots at *s*_min_<1 μeV). In addition, recent works have suggested that for InGaAs QDs entanglement is robust and the violation of the classical limit can be achieved for FSS smaller than 3–4 μeV (refs 37, 39). To quantify this probability in our ST-ELEDs, we use the following approach: we study the evolution of the fidelity to the maximally entangled Bell state |Ψ^+^〉 as a function of the FSS for one single QD and we use the obtained result to estimate at which value of the FSS it is possible to overcome the classical limit. To determine the entanglement fidelity, polarization correlations were performed for each value of the FSS and entanglement was equivalently quantified by measuring the degree of correlation (see Methods). As shown in Fig. 4b, the maximum fidelity *f*^+^=0.75±0.02 is achieved when the FSS is tuned close to zero. For FSS values larger than 3 μeV, the fidelity drastically decreases below the classical limit (see the dashed line). Taking into account that the exciton lifetime of the InGaAs QDs in our ST-ELED device has typical values of about 1 ns, this FSS of about 3 μeV provides an upper limit to observe entanglement, consistent with previous reports for InGaAs QDs^12^,^27^,^37^,^39^. From the statistical investigation we find that 27 QDs can be tuned below 3 μeV, which indicates a probability as high as 33% of QDs that can be exploited as entangled-light emitters in our ST-ELEDs. Compared with the only work on ELEDs present in the literature^16^, the yield demonstrated in our work is more than an order of magnitude higher (a factor of ∼30). This probability is higher than what was reported for highly symmetric pyramidal QDs where, however, electrical injection has not been realized yet^40^. We note that the yield of dots tuned for entangled-photon generation in our ST-ELED can be further improved by optimizing the alignment of the strain principal axes to the statistical mean value of the initial polarization direction. According to our statistical measurement, the mean value of the initial polarization direction is in fact 92.25° instead of 90° (see Supplementary Fig. 2).

### High speed generation of entangled-photon pairs

**Figure 5:**
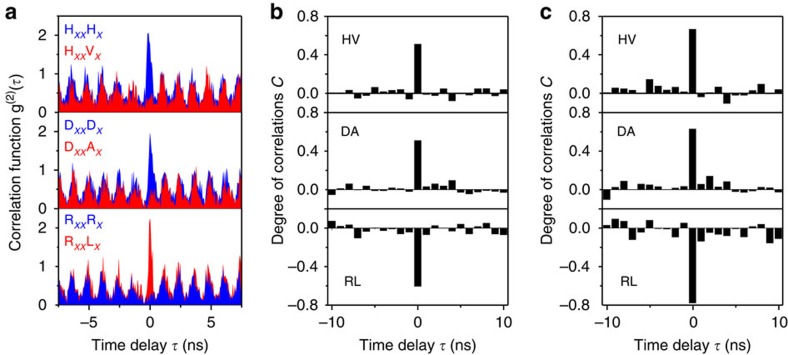
Polarization correlation results from the ST-ELED under electrically pulsed injection at repetition rate of 400 MHz. (**a**) Normalized correlation functions for co- and cross-polarized *XX* and *X* photons in HV, DA and RL bases. (**b**,**c**) Degree of correlation *C* in given basis, in which correlation in HV and DA bases (*C*>0) and anti-correlation (*C*<0) in RL basis are obtained without temporal gate (Δ*τ*=2.5 ns) and with a temporal gate width Δ*τ*=0.8 ns centred at 0 delay time, respectively.

In addition, we can increase the pulsed excitation rate to achieve fast generation rate of the entangled-photon pairs. This feature is highly desirable for high data-rate quantum information processing. Figure 5 shows the results of polarization correlation measurements at 400 MHz for the dot E. Similar to the case of 185.2 MHz, we observe correlations in the HV and DA bases and anti-correlation in the RL basis for co-polarized two photons. In Fig. 5b the degrees of correlation in given bases are reported. We find a state fidelity as high as 0.66±0.02, which exceeds the classical limit of 0.5 and thus proves generation of entangled-photon pairs at 400 MHz. We observe that the fidelity at 400 MHz is smaller than the fidelity at 185.2 MHz reported above. This is likely ascribed to the contribution of a small amount of uncorrelated photon pairs due to the time-dependent re-excitation process, residual FSS and background emission^16^,^38^,^39^. By temporal post selection of the emitted photons we can alleviate these effects for the entanglement degradation. The curves in Fig. 5c show the degrees of correlation for a temporal gate Δ*τ*=0.8 ns at which ∼20% of the coincidence counts are discarded. We measure *C*_HV_=0.67±0.06, *C*_DA_=0.63±0.04 and *C*_RL_=−0.78±0.07, corresponding to *f*^+^=0.77±0.03. The degree of correlation and entanglement fidelity can be improved by further shortening Δ*τ*^16^,^13^,^38^,^39^. With the narrowest available gate width of 0.1 ns applied, ∼80% coincidence counts are discarded and the degrees of correlation increase significantly: *C*_HV_=0.74±0.12, *C*_DA_=0.74±0.09 and *C*_RL_=−0.84±0.12, which provides the highest fidelity of 0.83±0.05. It is interesting to investigate whether such a high level of entanglement is sufficient to violate Bell’s inequality. Using the measured values of the degree of polarization correlation, it is possible to determine Bell parameters *S*_RD_, *S*_RC_ and *S*_DC_, which are related to three different planes of the Poincaré sphere (see Methods). Our results show that the Bell parameters increase as the gate width is decreased, and the Bell inequality is found to be violated starting from the gate width of 0.8 ns. From the degrees of correlation at Δ*τ*=0.8 ns, we calculate *S*_RD_=1.83±0.07, *S*_RC_=2.04±0.09 and *S*_DC_=2.00±0.08. Two of these values are >2 and they indicate violations of the Bell inequality. In particular, *S*_RD_ is found to be less than *S*_RC_ and *S*_DC_, and this is due to the weaker degree of correlation observed in the HV and DA bases (*C*_HV_, *C*_DA_<|*C*_RL_|) (refs 16, 37, 39). This common feature is usually observed for the QDs entangled-photon sources and is most probably ascribed to the weak coupling between the two bright exciton states^41^. Therefore, for the actual entangled state *S*_RD_ is not optimally chosen to inspect violation of the Bell inequality. In addition, for the gate width of 0.1 ns, we find *S*_RD_=2.09±0.21, *S*_RC_=2.23±0.21 and *S*_DC_=2.23±0.24 (see Supplementary Fig. 4). All these three parameters are above the threshold of 2, thus proving that our ST-ELED is capable of generating non-local states of light in response to an electrical trigger.

## Discussion

We have presented ST-ELEDs in which anisotropic strain fields are used to tune QDs for entangled-photon generation. We have shown that up to 30% of QDs embedded in this device are capable of emitting polarization entangled-photon pairs. This practically removes the tedious search for special QDs plaguing previous ELEDs^16^. Furthermore, we demonstrate triggered entangled-photon emission at a repetition rate of 400 MHz. Our all electrically controlled ST-ELEDs emerge as one of the most practical entangled-photon sources with fast operation speed and great potential for large-scale quantum communication and computation tasks.

Despite the considerable advances in our ST-ELED, it should be noted that the high yield of QDs tuned for entangled-photon emission has been achieved by applying different magnitudes of strain to different QDs in the single ST-ELED device due to the structural randomness of the QDs. To achieve scalable on-chip integrative entangled-photon applications, active engineering efforts are required to fabricate well-defined microstructures on PMN-PT crystal so that different strain fields can be exerted to different QDs simultaneously on one single chip. This will be of particular interest for realizing QDs arrays of independently tunable electrically triggered entangled-photon sources. Further improvements to the ST-ELED devices, such as integration with microcavities^42^,^43^ or micro-lenses^44^, to achieve bright entangled-photon emission, using III-nitride QDs to develop entangled-photon sources operating at room temperature^45^,^46^, constitute important steps towards realizing more practical electrically driven entangled-photon sources for scalable quantum information applications.

## Methods

### Sample and device fabrication

The studied sample was grown on a (001) GaAs substrate by solid-source molecular beam epitaxy. It consisted of a *p*-*i*-*n* heterostructure diode composed of a 178-nm-thick *n*-type GaAs layer, a 160-nm-thick intrinsic GaAs layer and a 96-nm-thick *p*-type GaAs layer from the bottom to the top. A layer of low-density (∼10^6^–10^7^ cm^−2^), self-assembled InGaAs QDs was embedded in the middle of the intrinsic GaAs layer. The entire diode structure was grown on a 100-nm-thick Al_0.75_Ga_0.25_As sacrificial layer. As for the device processing, first of all, standard ultraviolet photolithography and wet chemical etching were used to fabricate mesa structures with size of 120 × 160 μm^2^. The longer edge of the GaAs membrane was processed along [110] crystal axis of GaAs and—during the transfer onto the piezoelectric actuator—was carefully aligned along the *y* axis of the PMN-PT actuator. It is worth noting that the bonded gold layer on the bottom formed a *p*-contact, whereas the *n*-type contact was formed by depositing a gold pad with size of 50 × 50 μm^2^ on the top of the nanomembrane.

### Electrically pulsed excitation and spectroscopic measurements

The EL is observed when the diode is biased with a DC voltage (*V*_d_) above −1.7 V; however, it is slightly different from one device to another due to the different bonding conditions. The pulsed electrical excitation is accomplished by superimposing an ultrafast electrical pulse stream onto a −1.6 V DC bias by using a broad bandwidth bias-Tee. The pulse stream used in this work has nominal duration of 300 ps and amplitude of *V*_pp_=−8.0 V. The large magnitude of the pulse stream used here is caused by the low pulse injection efficiency, which is probably ascribed to the imperfect electrical connections and large impedance mismatching between the device and the external electronics. Further improvements, including optimization of the electrical connections and introducing an impedance matching network^47^, are expected to increase the pulse injection efficiency and consequently reduce the pulse magnitude.

In optical measurements, the EL emitted from the diode is collected by a × 50 microscope objective with numerical aperture of 0.42, which is placed on the top of the nanomembrane and collects the photon emission from the area close to the metal contact. By inserting a half-wave plate and a linear polarizer directly after the collection lens, polarization-resolved measurements were performed to obtain the FSS against *F*_p_. The exciton polarization is determined by aligning the fast optical axis of the polarizer along [110] direction of the nanomembrane. The EL was directed to a spectrometer with 750 mm focus length and the spectrum was analysed using a nitrogen-cooled charge-coupled device. The FSS is determined with an accuracy of sub-μeV by taking the experimental procedure in refs 23, 25.

### Polarization-resolved photon correlation measurements

Regarding the polarization-resolved correlation measurements, a non-polarizing 50:50 beam splitter is placed directly after the collection objective, to divide the optical paths between two spectrometers, which are used to detect *X* and *XX* separately. After each spectrometer, a Hanbury–Brown Twiss setup consisting of a polarizing beam splitter and two high-efficiency single-photon avalanche detectors is placed. Half and quarter waves were used to select the proper polarization basis. The temporal resolution of the system is ∼400 ps. The entanglement can be equivalently quantified by measuring degree of correlation C, which is defined by




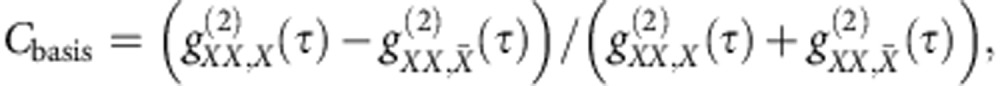




where *g*_*XX*,*X*_^(2)^(*τ*) and 
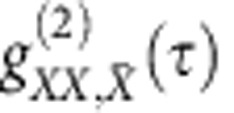
 are normalized second-order time correlations for co-polarized and cross-polarized *XX* and *X* photons, respectively. The fidelity *f*^+^ is calculated by using the formula: *f*^+^=(1+*C*_HV_+*C*_DA_−*C*_RL_), in which *C*_HV_, *C*_DA_ and *C*_RL_ are degree of correlations in HV, DA and RL bases. The Bell parameters are determined with the formulas: 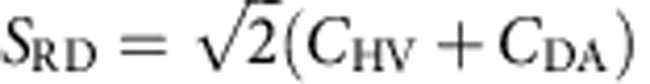, 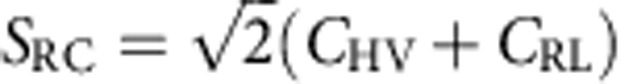 and 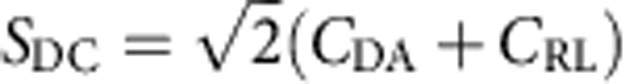.

## Additional information

**How to cite this article:** Zhang, J. *et al.* High yield and ultrafast sources of electrically triggered entangled-photon pairs based on strain-tunable quantum dots. *Nat. Commun.* 6:10067 doi: 10.1038/ncomms10067 (2015).

## Supplementary Material

Supplementary Information Supplementary Figures 1-4, Supplementary Tables 1-2, Supplementary Notes 1-3 and Supplementary References.
