# Corrigendum: High yield and ultrafast sources of electrically triggered entangled-photon pairs based on strain-tunable quantum dots

**DOI:** 10.1038/ncomms11681

**Published:** 2016-05-11

**Authors:** Jiaxiang Zhang, Johannes S. Wildmann, Fei Ding, Rinaldo Trotta, Yongheng Huo, Eugenio Zallo, Daniel Huber, Armando Rastelli, Oliver G. Schmidt

Nature Communications 6: Article number: 10067 10.1038/ncomms10067 (2015); Published: 12012015; Updated: 05112016

This Article contains errors in the labelling of the ticks of the *x* axis in Fig. 5. In panel a, the *x* axis values should have been labelled ‘−10 ns' and ‘10 ns' rather than ‘−5 ns' and ‘5 ns'. In panels b and c, the *x* axis values should have been labelled ‘−25 ns' and ‘25 ns' rather than ‘−10 ns' and ‘10 ns'. The correct version of this figure appears below.

[Fig i1]

## Figures and Tables

**Figure i1:**
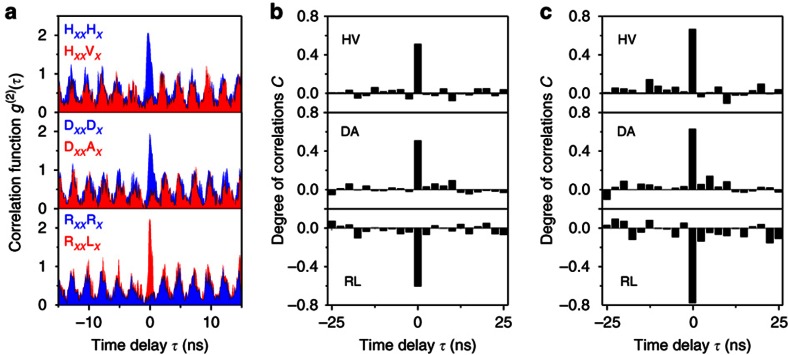
**Figure 5 | Polarization correlation results from the ST-ELED under electrically pulsed injection at repetition rate of 400 MHz.** (**a**) Normalized correlation functions for co- and cross-polarized XX and X photons in HV, DA and RL bases. (**b,c**) Degree of correlation C in given basis, in which correlation in HV and DA bases (C>0) and anti-correlation (C<0) in RL basis are obtained without temporal gate (Δ*τ*=2.5 ns) and with a temporal gate width Δ*τ*=0.8 ns centred at 0 delay time, respectively.

