# Supplementary material for: High yield and ultrafast sources of electrically triggered entangled-photon pairs based on strain-tunable quantum dots
Source: Nat Commun. 2015 Dec 1;6:10067. doi: 10.1038/ncomms10067 (PMC4686767; doi:10.1038/ncomms10067)
Supplement: Supplementary Information — Supplementary Figures 1-4, Supplementary Tables 1-2, Supplementary Notes 1-3 and Supplementary References. [file ncomms10067-s1.pdf]

Supplementary Figures

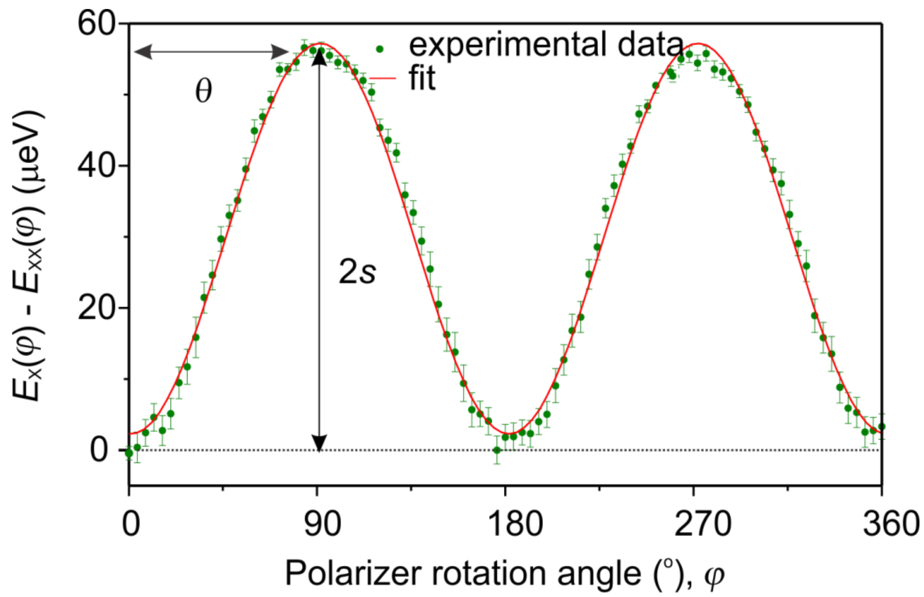

Supplementary Figure 1: Experimental measurement of the fine structure splitting (FSS) and polarization angle (θ). $E_X(\varphi)$ - $E_{XX}(\varphi)$ is obtained from a polarization-resolved measurement at a given strain field (or F_p) after subtracting E_0 = 0.00274 eV (where E_0 represents the minimum value of the difference between exciton and biexciton energy). φ is the polarization direction selected by the polarization analyzer which varies from 0° to 360°. From this measurement, we can extract the FSS (s) and the polarization angle of high-energy component of the exciton emission θ at different strain fields. The initial FSS (s_0) and polarization angle (θ_0) are defined at F_p = 0 kV cm^{-1}. Note that the error is defined as the standard deviation and the same definition is applied below.

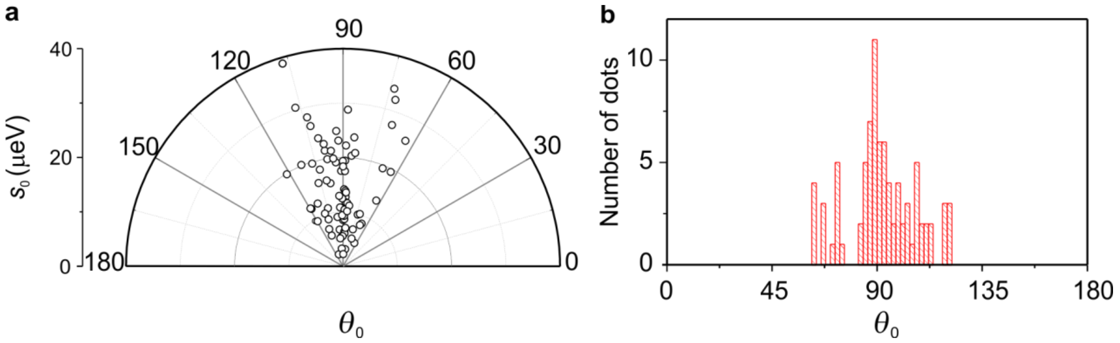

Supplementary Figure 2: Statistics of θ_0 and s_0. (a) Scatter plot of the polarization angle θ_0 with respect to the [110] direction of the GaAs at zero strain fields . The data is obtained by characterizing 82 randomly selected QDs in our S-ELED device and each dot has distinct exciton and biexciton emission. It is clearly seen that the majority of dots have θ_0 at about 90°, that is, the [1-10] crystal axis of GaAs. Some scatter of θ_0 in a range of 60° - 120° has also been observed which is likely due to the anisotropy in strain and composition formed during the growth process. Meanwhile, a large number of dots have small deviations from the [1-10] crystal axis, which can provide a tolerance to the alignment of the strain direction with respect to the crystal axes. Our statistical investigation presented here is in line with the results reported in ref. 1. In addition, the initial FSS s_0 was revealed to have average value of about 20 μeV, and the dots with sufficient small s_0 for entanglement are barely found without applying any strain fields. (**b**) Statistical measurent reveals that the mean value of the initial polarization angle is 92.25° and the standard deviation is 14.41° .

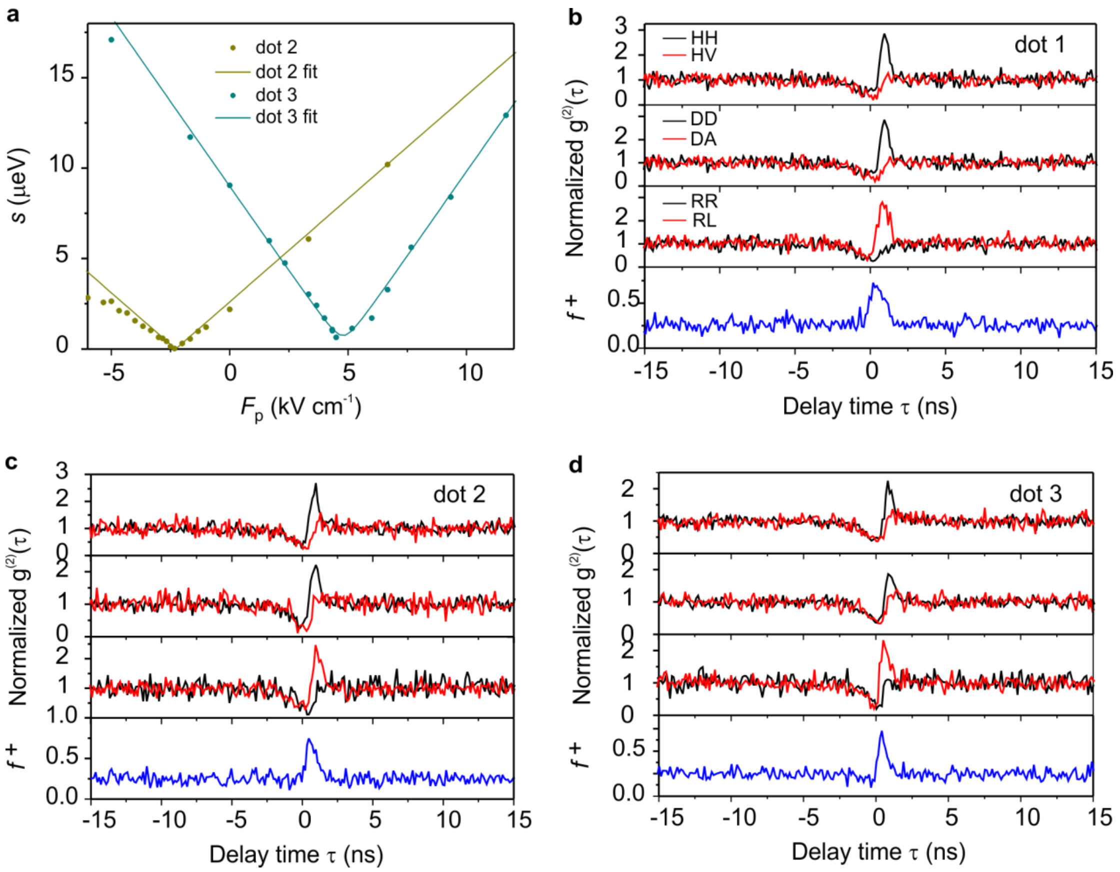

Supplementary Figure 3: Entanglement results for other three dots. (a) *s* as function of the electric field F_p applied on the PMN-PT for the dot 2 and dot 3, respectively. **(b)** – **(d)** The polarization correlation results measured in rectilinear (HV), diagonal (DA) and circular (RL) bases for the dot 1, 2, 3. It is clearly seen that, for co-polarized exciton and biexciton photons (black curves), correlation is always observed in HV(DA) basis and anti-correlation in RL basis. The fidelity (f^+) is found to be 0.71±0.03, 0.75±0.02 and 0.71±0.02 for the dot 1, 2 and 3, respectively.

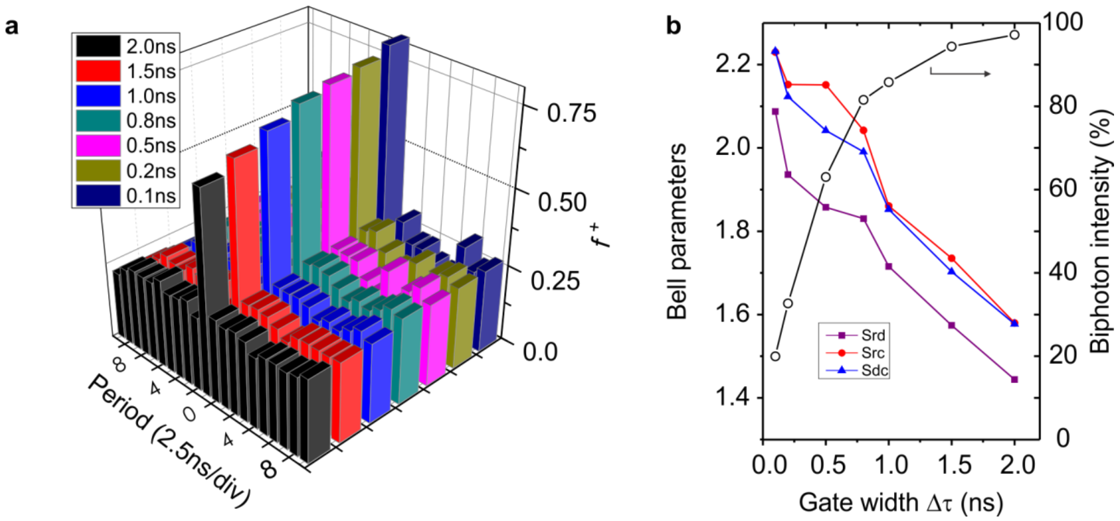

Supplementary Figure 4: **Time gating and violation of Bell's inequality. (a)** f^+ as a function of the temporal gate width, which is extracted from the co- and cross-polarization correlations at the excitation repetition rate of 400 MHz as shown in Fig. 5a in the main text. By narrowing the gate width from 2.0 to 0.1 ns, the fidelity increases drastically from 0.67±0.02 to 0.83±0.05. **(b)** Gate width ($\Delta\tau$) dependent change of Bell parameters (S_{RD}, S_{RC} and S_{DC}) and the biphoton intensity. In our experiment, we can clearly see that all the three Bell parameters increase rapidly as the gate width decreases. S_{RD} is found smaller than S_{RC} and S_{DC} due to the effect of the fluctuating magnetic fields produced by the QD nuclei, as pointed out by Stevenson et al. [2] Therefore, for actual entangled state, S_{RD} is not necessarily optimized to test Bell's inequality [3, 4]. We found that S_{RC} and S_{DC} become larger than 2 as the gate width is narrowed down to 0.8 ns, while for a gate width of 0.1 ns all the three Bell's

parameters shown violation of Bell's inequality by at least one standard deviation. Furthermore, the proportion of the total biphoton intensity as a function of the time gate width is also shown. Although the fidelity and the Bell parameter increase as we decrease the gate width, we also note a drastic decrease of the biphoton intensity as the gate width is decreased. At the gate width of 2.5 ns, all the coincidence counts are included for calculating the degree of correlation (see in the main text) and thus it corresponds to a biphoton intensity proportion of 100%. As smaller gate width is applied from 2.0 ns to 0.1 ns, the biphoton intensity is reduced quickly from 97% to 16%.

Supplementary Tables

Supplementary Table 1: Summary of s_0, θ_0, k, δ for the studied five dots.

QDs	θ_0 (^{0})	s_0 (μeV)	κ (μeV)	δ (μeV)
A	102.0±0.4	20.1±0.2	4.11±0.1	-9.20±0.08
B	92.7±0.2	34.9±0.2	1.60±0.08	-17.4±0.1
C	86.1±0.4	17.31±0.2	-1.20±0.05	-8.5±0.1
D	90.4±0.3	16.9±0.1	0.12±0.05	-8.40±0.07
E	90.6±0.3	27.4±0.3	0.28±0.07	-13.7±0.1

Supplementary Table 2: Summary of entanglement results for other three dots.

dots	X (eV)	XX(eV)	V_d (V)	s_{min} (μeV)	f^+
1	1.4127	1.4122	-1.75	0.30 ±0.25	0.72±0.03
2	1.4013	1.4000	-1.98	0.20±0.30	0.75±0.02
3	1.4000	1.3993	- 2.05	0.60±0.20	0.71± 0.02

The binding energy of exciton and biexciton were measured at F_p = 0 kV cm^{-1} and all the dots are characterized under d. c. current injection.

Supplementary Notes

Supplementary Note 1: Derivation of the volumetric strain due to anisotropic biaxial stress

According to the deformation potential theory, the strain-induced-change in the energy bandgap of a semiconductor (and hence the emission energy of a quantum dot) mostly depends of the volumetric strain (i. e. the trace of the strain tensor) [6]. The expression for the volumetric strain $\varepsilon_{\mathrm{tot}} = \varepsilon_{\mathrm{xx}} + \varepsilon_{\mathrm{yy}} + \varepsilon_{\mathrm{zz}}$ for purely in-plane stress (as in our experiments) can be obtained using the relation between the normal stress and strain components for cubic materials:

$$\begin{pmatrix} \varepsilon_{\mathrm{xx}} \\ \varepsilon_{\mathrm{yy}} \\ \varepsilon_{\mathrm{zz}} \end{pmatrix} = \begin{pmatrix} S_{11} & S_{12} & S_{12} \\ S_{12} & S_{11} & S_{12} \\ S_{12} & S_{12} & S_{11} \end{pmatrix} \begin{pmatrix} \sigma_{\mathrm{xx}} \\ \sigma_{\mathrm{yy}} \\ 0 \end{pmatrix} \quad (1)$$

From this expression we can obtain two equations:

$$\varepsilon_{\mathrm{tot}} = \varepsilon_{\mathrm{xx}} + \varepsilon_{\mathrm{yy}} + \varepsilon_{\mathrm{zz}} = (S_{11} + 2S_{12})(\sigma_{\mathrm{xx}} + \sigma_{\mathrm{yy}}) \quad (2)$$

$$\varepsilon_{\mathrm{xx}} + \varepsilon_{\mathrm{yy}} = (S_{11} + S_{12})(\sigma_{\mathrm{xx}} + \sigma_{\mathrm{yy}}). \quad (3)$$

From eq. 3 we can obtain the total stress $\sigma_{\mathrm{xx}} + \sigma_{\mathrm{yy}}$ as a function of $\varepsilon_{\mathrm{xx}} + \varepsilon_{\mathrm{yy}}$. By combining the result with the first equation we obtain the equation provided in the main text:

$$\varepsilon_{\mathrm{tot}} = \varepsilon_{\mathrm{xx}} + \varepsilon_{\mathrm{yy}} + \varepsilon_{\mathrm{zz}} = (S_{11} + 2S_{12})(\sigma_{\mathrm{xx}} + \sigma_{\mathrm{yy}}) = \frac{S_{11}+2S_{12}}{S_{11}+S_{12}}(\varepsilon_{\mathrm{xx}} + \varepsilon_{\mathrm{yy}}) = \frac{S_{11}+2S_{12}}{S_{11}+S_{12}}(1-0.37)\varepsilon_{\mathrm{xx}}. \quad (4)$$

Note that the same result is obtained independent on the choice of the *x* and *y* axes ([110] and [1-10] as in the text or [100] and [010]), as it can be verified explicitly by a rotation of the strain tensor.

Supplementary Note 2: FSS and exciton polarization direction θ: theoretical background

The effect of our anisotropic stress applied to the QDs can be seen as two stresses p_{xx} and p_{yy} along the [1-10] and [110] crystal directions, respectively. Note that the behaviors of FSS (s) are almost symmetric for stresses along the [110] and [1-10] directions; i. e., the effects of tensile stress along the [110] direction are almost identical to the effects of compression along the [1-10] direction [6]. Therefore, our anisotropic stress can be reliably treated as a 'uniaxial stress' along one of the principal stress axes ([110] or [1-10] direction). The behavior of s and θ can be described using the following formulas [6]:

$$s = \sqrt{4(\beta p + k)^2 + (\alpha p + 2\delta)^2} \quad (5)$$

$$\theta = tan^{-1}\left(\frac{-2\delta - p\alpha \pm s}{2(\beta p + k)}\right)$$

(6)

Where p is the magnitude of the external stress and it is determined by $p = p_{\mathrm{yy}} - p_{\mathrm{xx}}$; β and α are stress dependent parameters, and k, δ depends on the mesoscopic structure of the QDs. For stress along either [110] or [1-10] direction, $\beta \approx 0$ and the

minimum FSS is determined by $s_{\min} = 2|k| = s_0 \times \sin(2\theta_0)$ (s_0 and θ_0 are the FSS and polarization angle at zero stress (see Supplementary Figure 1). In addition, k, δ can be experimentally determined by $k = -s_0 \times \sin(2\theta_0)/2$, $\delta = s_0 \times \cos(2\theta_0)/2$. Supplementary Table 1 is a summary of s_0, θ_0, k, δ for the five studied dots in the main text. With these parameters, we can fit the experimental data theoretically using formulas (5) and (6) above, as the solid lines show in Fig. 2 in the main text.

Supplementary Note 3: Entanglement analysis for other dots at $s_{\min}$ < 1 μeV

In addition to the QD E characterized in the main text, here we present an entanglement characterization for additional three dots. Supplementary Table 2 summarizes the entanglement results for these other dots in terms of the fidelity to the maximally entangled Bell state $|\Psi^+ \rangle = 1/\sqrt{2}\,(|\mathrm{H}_{XX}\mathrm{H}_\mathrm{X}\rangle + |\mathrm{V}_{XX}\mathrm{V}_X\rangle)$. The dot 1 is the dot D studied in the main text and its strain tuning behavior is shown in Fig. 2a and 2b (red curves). The change of the FSS with the external strain fields for the other two dots is shown in Supplementary Figure 3. By measuring the degree of correlation in given basis at the minimum FSS, the fidelity of these dots is found to be 0.72±0.03, 0.75±0.02 and 0.71±0.02 respectively. Such high values of fidelity exceed the classical limit of 0.5 and suggest all these dots have been successfully tuned to be 'good' entangled light sources via the external strain field.

Supplementary References

1. Bennett, A. J. et al. Electric-field-induced coherent coupling of the exciton states in a single quantum dot. *Nat. Physics* **6**, 947 – 950 (2010).
2. R. M. Stevenson. et al. Preprint at http://arxiv.org/abs/1103.2969 (2011).
3. R. Trotta, et al. Highly Entangled Photons from Hybrid Piezoelectric-Semiconductor Quantum Dot Devices. *Nano Lett.* **14**, 3439 – 3444 (2014).
4. Salter, C. L., Stevenson, R. M., Farrer, I., Nicoll, C. A., Ritchie, D. A. & Shields, A. J. An entangled-light-emitting diode. *Nature* **465,** 594–597 (2010).
5. Chuang, S. L. *Physics of Optoelectronic Devices*. (Wiley New York, 1995).
6. Gong, M., Zhang, W., Guo, G.-C. & He, L. X. Exciton Polarization, Fine-Structure Splitting, and the Asymmetry of Quantum Dots under Uniaxial Stress. *Phys. Rev. Lett.* **106,** 227401 (2011).
